# Asymptomatic intracardiac mass in a 14-year-old girl with granulomatosis with polyangiitis: Case report

**DOI:** 10.1186/1546-0096-10-9

**Published:** 2012-04-13

**Authors:** Julia G Harris, David M Salvay, Marisa S Klein-Gitelman

**Affiliations:** 1 Medical College of Wisconsin, Children’s Hospital of Wisconsin, Milwaukee, WI, USA; 2 The University of Chicago, NorthShore University HealthSystem, Evanston, IL, USA; 3 Northwestern University, Children’s Memorial Hospital, Chicago, IL, USA; 4 Children’s Hospital of Wisconsin, 9000 West Wisconsin Avenue, Wauwatosa, WI, 53226, USA; 5 The University of Chicago, NorthShore University HealthSystem, Evanston Hospital, 2650 Ridge Avenue, Room #5319, Evanston, IL, 60201, USA; 6 Children’s Memorial Hospital, 2200 Children’s Plaza, Chicago, IL, 60614, USA

**Keywords:** Granulomatosis with polyangiitis, Wegener’s granulomatosis, Intracardiac mass

## Abstract

Granulomatosis with polyangiitis (GPA; Wegener’s granulomatosis) is a systemic necrotizing vasculitis of unknown etiology that commonly involves the upper airways, lungs, and kidneys. Cardiac involvement with an intracardiac mass is an exceedingly rare manifestation of this disease, especially in the pediatric population where, to our knowledge, only one article exists to date that has described such a finding. In this report, we present the case of an adolescent female who initially presented with renal failure and an intracardiac mass. Subsequent work-up led to a diagnosis of granulomatosis with polyangiitis (GPA). Cardiac manifestations in pediatric GPA are not common; however, they may be more prevalent than reported given recent adult literature and concern for clinically silent abnormalities.

## Background

Granulomatosis with polyangiitis (GPA; Wegener’s granulomatosis) is a systemic vasculitis characterized by acute necrotizing granulomas of the upper respiratory tract and lungs, necrotizing or granulomatous vasculitis affecting small to medium-sized vessels and pauci-immune, necrotizing, and crescentic glomerulonephritis; however, this disease can affect virtually any organ system [1-7]. In this report, we describe a case of GPA presenting as renal failure with discovery of an intracardiac mass in an adolescent patient. We will discuss the significance of this finding in light of recent literature.

## Case Presentation

A 14-year-old girl presented with a 6-week history of fatigue, persistent rhinorrhea, and intermittent epistaxis. Her history was also significant for recurrent sinusitis, rash, decreased urine output, and myalgias. The patient was seen by her pediatrician with concerns that her nose was “collapsing.” Past medical history includes atopic dermatitis as a child and allergic rhinitis.

On admission to our hospital, the patient’s physical exam was remarkable for pallor, saddle nose, dried nasal secretions, oral ulcers, synovial swelling around multiple joints, and palpable purpura on her distal extremities. Initial laboratory tests showed that the patient was in acute renal failure with a BUN of 131 mg/dL and creatinine of 11.14 mg/dL. She also had elevated inflammatory markers (ESR 82 mm/hr, CRP 19.2 mg/dL), normocytic anemia (hemoglobin 8.7 g/dL, MCV 79.7 fL), leukocytosis (white blood cells 29 × 10^3^/mm^3^, absolute eosinophil count 615/uL), thrombocytosis (platelets 661 × 10^3^/mm^3^), and proteinuria and hematuria on urinalysis (specific gravity 1.015, pH 6.0, large occult blood, RBC 21–30/hpf, protein 100 mg/dL, trace leukocytes, WBC 0–2/hpf, epithelial cells 6–10/hpf; negative nitrite, bilirubin, ketones, and glucose). Initial imaging studies showed a left lower lobe infiltrate via chest x-ray and renal ultrasound demonstrated large, echogenic kidneys with absent corticomedullary junction differentiation.

**Figure 1:**
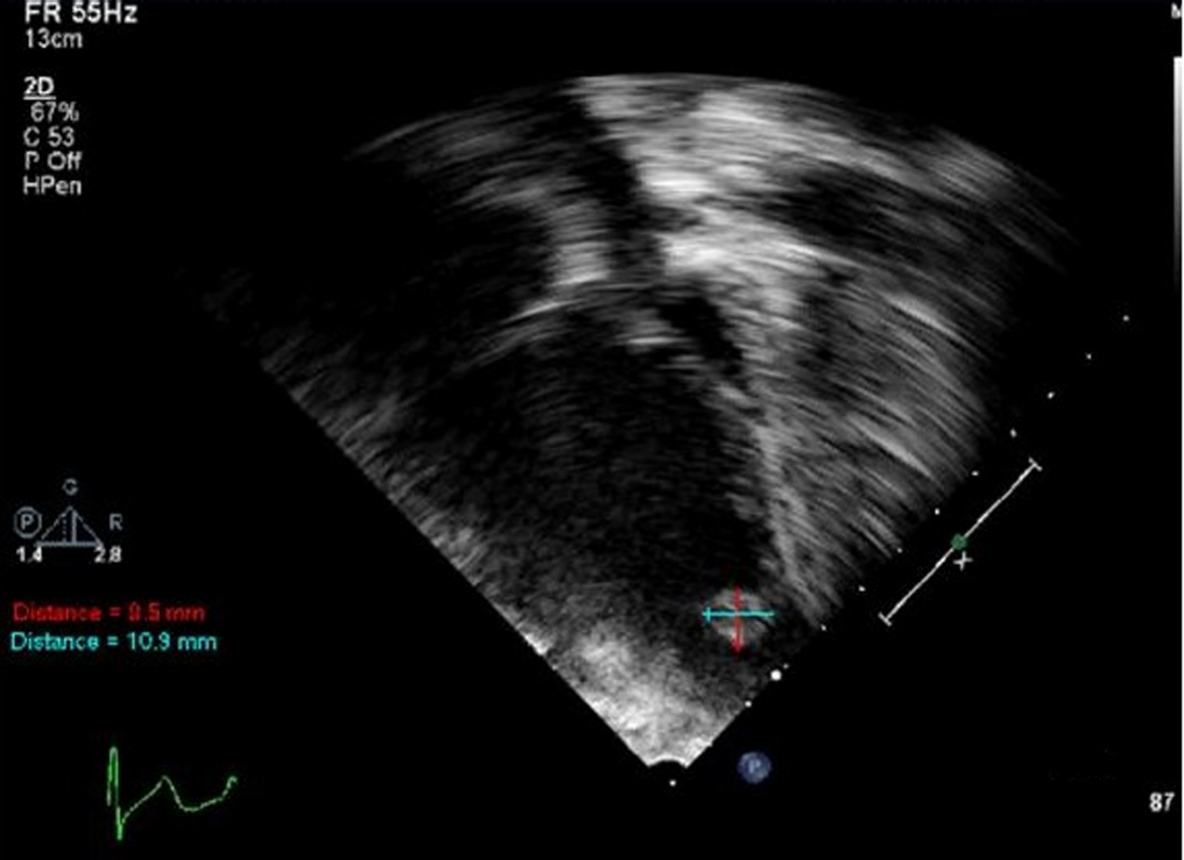
**Transthoracic 2D echocardiogram.** Apical four chamber view demonstrating a pedunculated mass near the apex of the left ventricle measuring 0.9 cm × 1.1 cm (arrows).

Given her vasculitic rash, nasal cartilage destruction, and apparent sinusitis—signs consistent with GPA—the patient was started on methylprednisolone (30 mg/kg/day) and cyclophosphamide (1 mg/kg/day) with decreased dosing secondary to renal failure. Additional laboratory results showed a positive ANCA with a cytoplasmic pattern by indirect immunofluorescence screening and an anti-proteinase 3 greater than 148 EIA/U by rapid enzyme immunoassay. On hospital day two, the patient became anuric and was started on hemodialysis. She was transferred to our intensive care unit due to hypoxia and dyspnea where she received positive pressure ventilation. In addition to hemodialysis, she underwent seven sessions of plasmapheresis. Due to worsening pulmonary symptoms, a bronchoscopy was performed which showed tracheal and bronchial ulcerations as well as pulmonary hemorrhage with the presence of bloody lavage fluid. Additionally, nasal endoscopy revealed nasal perforations with necrotic mucosa. A biopsy of her nasal mucosa demonstrated acute inflammation and necrosis without evidence of granulomatous vasculitis. A kidney biopsy found diffuse pauci-immune necrotizing and crescentic glomerulonephritis with necrotizing arteriolitis and thrombotic microangiopathy. Surprisingly, an echocardiogram done pre-operatively and prior to the previous procedures was significant for a large ovoid, homogeneous mass in the apex of the left ventricle (Figure 1). At the same time, she was noted to have decreased systolic function with an ejection fraction of 40% and was incidentally found to have a bicuspid aortic valve. Due to concern over her decreased cardiac function, the patient was started on milrinone for inotropic support.

**Figure 2:**
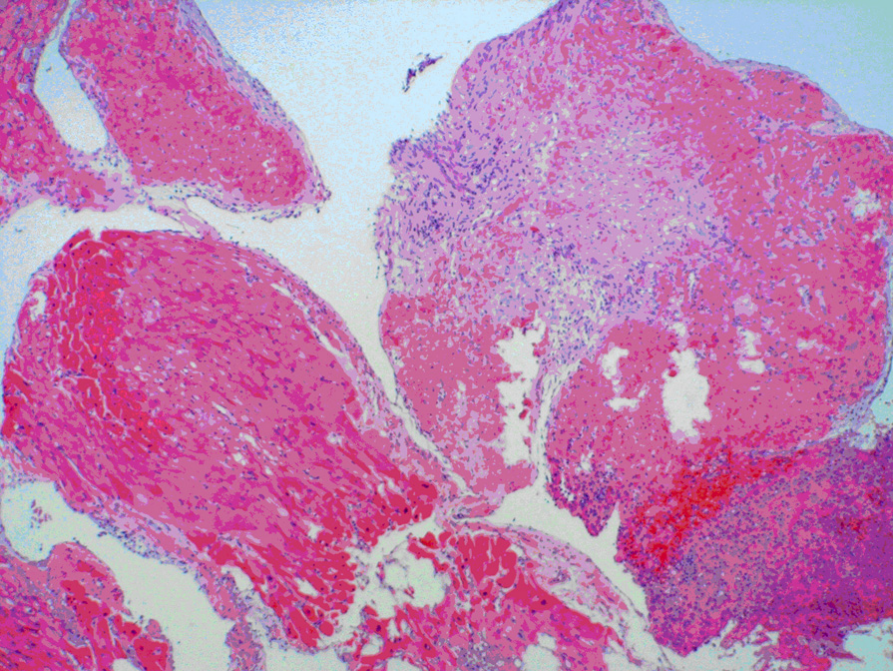
Fragments of acutely inflamed myocardium with organizing granulation tissue and adjacent fibrino-purulent material.

Cardiovascular Surgery was consulted for removal of the intracardiac mass since its location and pedunculated character placed the patient at significant risk for embolic complications. Prior to surgery, anticoagulation with aspirin (325 mg/day) was initiated, and a cardiac MRI was obtained to further analyze the mass and evaluate for other cardiac changes. MRI showed a pedunculated left ventricle mass (1.45 cm × 0.95 cm), global hypokinesis, and depressed left ventricular function. Cardiovascular Surgery performed a sternotomy followed by resection of the mass via a trans-aortic approach. Intra-operative findings were notable for a 1.1 cm × 0.8 cm × 0.5 cm soft, tan-white, tissue mass adherent to ventricle trebeculations. An endomyocardial biopsy was also obtained. Pathology of the left ventricular mass showed necrotic tissue with acute inflammation consisting of neutrophils and eosinophils with absence of granulomas (Figure 2).

Following the operation, the patient had an episode of non-sustained ventricular tachycardia with manipulation of pacer wires that remained post-operatively. A follow-up echocardiogram on post-operative day two demonstrated low-normal left ventricular systolic function (shortening fraction 32%) and mild aortic and mitral valve regurgitation.

Cyclophosphamide was continued at 1 mg/kg/day and methylprednisolone at 2 mg/kg/day. Trimethoprim/sulfamethoxazole was also added for Pneumocystis prophylaxis. Prior to discharge, the patient had no further cardiac issues and started to produce small amounts of urine but remained in clinical renal failure. After twenty-one days in the hospital, she was discharged home with plans for hemodialysis three days per week.

## Discussion

Granulomatosis with polyangiitis can affect virtually every organ system in the body but primarily targets the upper airways, lower respiratory tract, and kidneys [1,3-9]. Clinically evident cardiac manifestations in GPA, especially in pediatric patients, are rare. A review of pediatric GPA by Akikusa *et al.* did not mention cardiac findings as a prominent clinical feature at presentation or during the follow-up period [4]. The ARCHiVe (A Registry for Childhood Vasculitis: e-entry) cohort also found no cardiovascular manifestations at presentation in 65 pediatric GPA patients [6]. However, Rottem *et al.* reported 39% of 23 patients with childhood-onset GPA had pericarditis at onset [7]. In adults, cardiac involvement in GPA has been reported in between <4% to 44% of cases and usually manifesting as pericarditis (50%), coronary arteritis (50%), myocarditis (25%), or valvulitis/endocarditis (21%) [2,8,10]. Recent reports in the adult literature suggest that cardiac involvement in small and medium vessel vasculitides may be more common than previously thought [11-13]. In fact, in a retrospective chart review of 85 patients with confirmed GPA, 86% were found to have echocardiographic abnormalities [13]. Another study found nine of eleven patients with GPA resistant to induction therapy had cardiac involvement based on transthoracic echocardiogram and cardiac MRI [9]. Abnormalities included all nine patients with late gadolinium enhancement lesions involving left ventricular myocardium, six patients with myocarditis, five patients with pericardial effusions, and two patients with regional wall motion abnormalities. The increase in the prevalence of cardiac involvement in adult GPA may be attributable to the proliferation and routine use of advanced imaging modalities such as cardiac MRI, echocardiography, and positron emission tomography in making the clinical diagnosis.

Intracardiac masses, however, remain extremely rare and there are only a few reports in the literature describing such findings in patients with GPA. Herbst *et al.* described a 56-year-old woman with chest pain and dyspnea who was found to have a mass involving the intracardiac septum and mitral valve who was subsequently diagnosed with GPA [14]. In another report, a 63-year-old woman presenting with syncope had GPA limited to her nasal mucosa but was later found to have a granulomatous mass involving the aortic outflow tract [15]. To the best of our knowledge, the only other report of a pediatric patient with GPA and an intracardiac mass was made by Kosovsky *et al.*[16]. They described a 16-year-old boy who presented with parasternal discomfort and ventricular tachycardia who was later found to have a mass involving the right ventricular wall with extension into the right ventricular outflow tract. Pathological examination of the resected mass revealed granulomatous vasculitis.

The development of an intracardiac mass may be influenced by regional wall abnormalities that have been found in high prevalence in younger patients with GPA as reported by Oliveria *et al.*[15].

## Conclusions

In conclusion, cardiac involvement in GPA is rare in pediatric patients. However, these manifestations should be considered in this patient population given the higher prevalence in adult patients and clinically silent cardiac abnormalities, such as an intracardiac mass, that may be present.

## Consent

Written informed consent was obtained from the patient and parent for publication of this case report and any accompanying images. A copy of the written consent is available for review by the Editor-in-Chief of this journal.

## Abbreviations

GPA, Granulomatosis with polyangiitis; MRI, Magnetic resonance imaging.

## Competing interests

The authors declare that they have no competing interests.

## Authors’ contributions

All authors participated in literature review, design, and drafting of the manuscript. In addition, all authors read and approved the final manuscript.
